# P-1987. Effectiveness of Tixagevimab-Cilgavimab as Pre-Exposure Prophylaxis in Patients Undergoing Hematopoietic Cell Transplant or Chimeric Antigen Receptor T-cell Therapy

**DOI:** 10.1093/ofid/ofae631.2145

**Published:** 2025-01-29

**Authors:** Arjun Juneja, Paul Armistead, Astha Thakkar, Jonathan Ptachcinski, Catherine A Herman

**Affiliations:** UNC School of Medicine, Chapel Hill, North Carolina; UNC Department of Medicine, Chapel Hill, North Carolina; UNC Department of Medicine, Chapel Hill, North Carolina; UNC Department of Pharmacy, Chapel Hill, North Carolina; UNC Eshelman School of Pharmacy, Chapel Hill, North Carolina

## Abstract

**Background:**

Tixagevimab-cilgavimab (tixa-cil), was approved for emergency use authorization by the FDA in December of 2021 for pre-exposure prophylaxis of the COVID-19 virus in immunocompromised populations due to concerns about vaccine efficacy. Data regarding real world efficacy of tixa-cil in preventing SARS-CoV-2 infection in immunocompromised patients is limited.

**Table 1:**
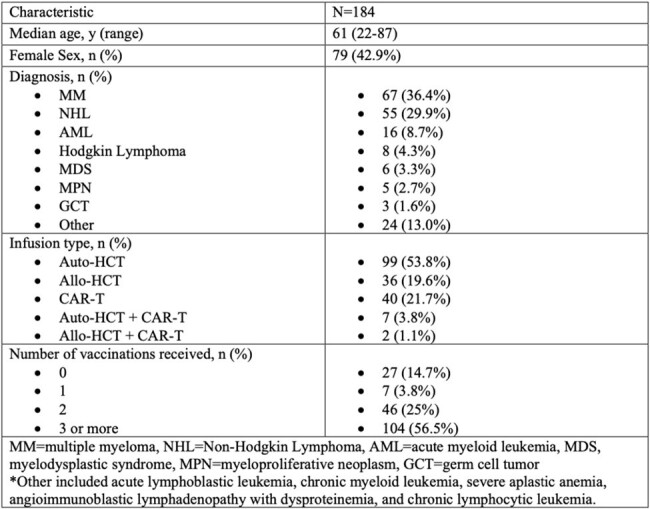
Patient Demographics

**Methods:**

We conducted a retrospective analysis of all patients who had received hematopoietic cell transplantation (HCT) or chimeric antigen receptor T-cell (CAR-T cell) therapy between 12/1/2021-12/1/2022 at the University of North Carolina Bone Marrow Transplant and Cellular Therapy Program and were eligible to receive tixa-cil as pre-exposure prophylaxis. Patient demographics, underlying malignancy type, COVID-19 vaccine status, data and number of tixa-cil injections, cellular infusion type, presence of GVHD, adverse events, and cause of death if related to COVID-19 were all collected.

**Table 2:**
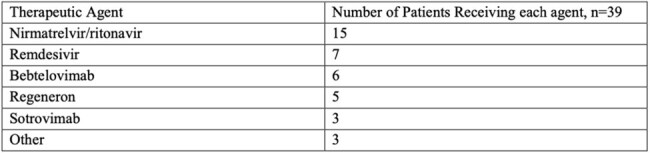
Therapeutic Agents Used For Treatment of COVID-19

**Results:**

Of 184 eligible patients, 113 (61%) received at least one tixa-cil injection. Forty-five cases of either documented or reported COVID-19 infection following cell infusion (including 8 recurrent infections) were identified. Of the 113 patients who received tixa-cil, 14 (12%) had a documented positive COVID test within 6 months. In comparison, 23 (32%) of the 71 patients who did not receive tixa-cel had a documented positive COVID test, (p=0.0013, Fisher’s exact test), OR=0.2951. Of 13 COVID-related hospitalizations, only one occurred in a patient who received tixa-cil, (p=0.0048, Fisher’s exact), OR=0.0641.

Number of Patients with Documented COVID Infections Who Did and Did Not Receive Tixa-Cil

**Figure:**
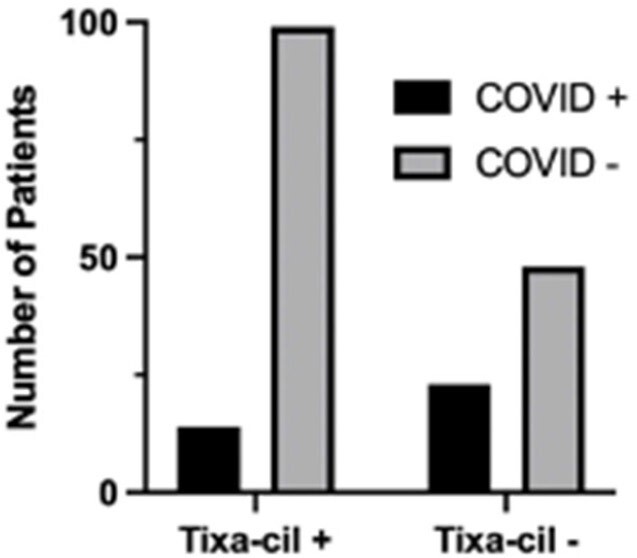

**Conclusion:**

Our findings indicate that tixa-cil was effective as pre-exposure prophylaxis in reducing SARS-CoV-2 infection and disease severity in a highly immunocompromised patient population. While the FDA removed authorization for ongoing use of tixa-cil in January 2023 due to its ineffectiveness against the Omicron variants, our data suggest that mAbs are highly effective in providing protective passive immunity to patients at risk of impaired vaccination responses. Pemibivart, a new anti-SARS-CoV-2 spike protein mAb, was recently authorized by the FDA. Our results suggest a clinical benefit for this therapy; however, real world studies will be needed to determine actual efficacy.

Number of Patients Hospitalized Related to COVID Infection Who Did and Did Not Receive Tixa-Cil

**Figure:**
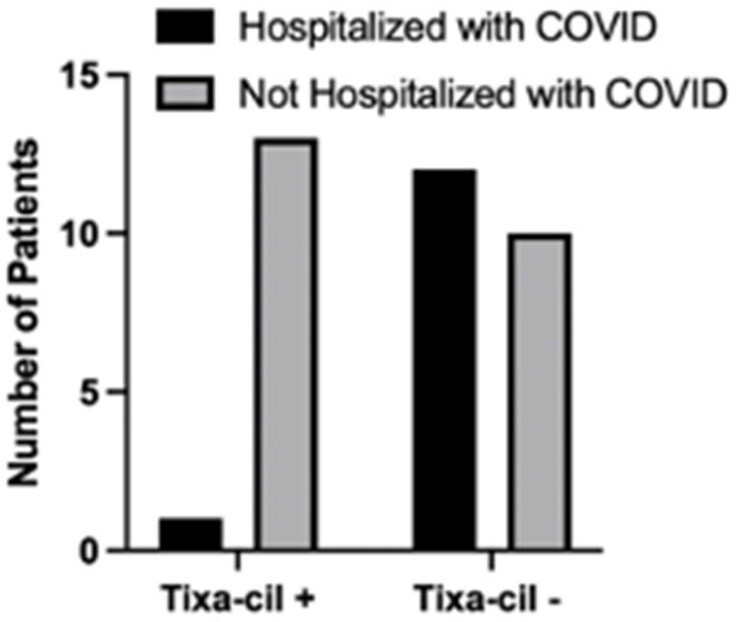

**Disclosures:**

Jonathan Ptachcinski, PharmD, Johnson & Johnson: Company Employee

